# Semiconductor Nanomaterials for Optoelectronic Applications

**DOI:** 10.3390/nano14231896

**Published:** 2024-11-26

**Authors:** Ikai Lo

**Affiliations:** Department of Physics, National Sun Yat-Sen University, Kaohsiung 80424, Taiwan; ikailo@mail.nsysu.edu.tw

## 1. Introduction

Nanotechnology has been comprehensively investigated for more than 30 years. A symbolic experiment of quantum corral was constructed through the atomic manipulation of 48 iron atoms on a copper surface via scanning tunneling microscopy to form a confinement potential barrier (corral) with a diameter of 14.26 nm. This experimentally demonstrated a concentric standing wave of quantum mechanics for electrons inside the corral with a diameter of 14 nm [1], indicating that quantum mechanics plays a key role in nanometer scale electron systems (quantum effect). For instance, in a Si-MOSFET device, a triangular confinement potential well is established by applying gate voltage above an insulated oxide layer to form a channel for current flow between the source and drain, and the depth of the confinement potential well is about 10 nm (depending on gate voltage) along *z*-axis away from the Si/oxide interface, which is enough to form a 2D electron gas (2DEG) with a series of quantized electron energy levels (i.e., 2D subbands: *E*_1_, *E*_2_, *E*_3_, …) inside the triangular potential quantum well (QW). The electron mobility of the 2DEG channel can achieve a huge enhancement by many orders of magnitude, simply by engineering a band profile or even by changing the host material to III-V heterostructured compounds [2,3,4]. Furthermore, Chenming Hu (U.C. Berkeley) et al. modified the 2D planar gate MOSFET to a 3D gate Fin Field Effect Transistor (FinFET) to improve the confinement potential well for a better switching time and higher current density [5]. However, when the size of the Fin-gated FET structure reduced down to 10 nm, a 1D nanowire channel expectedly opened between source and drain, with a series of quantized electron energy levels of 1DEG (i.e., 1D subbands). Recently, the size of the FinFET structure was even narrowed down to 1.8 nm by Taiwan Semiconductor Manufacturing Company (TSMC) using DUV lithography (1.8 nm = 18 angstroms, which is also named 18A technology), a top-down approach to nanotechnology that challenges Golden Moore’s Law. Moreover, a series of discrete virtual atomic-like electron energy levels (i.e., 0DEG electron system) can be created using a splitting gate on 2D planar semiconductor QW, a so-called quantum dot (QD) structure [6].

What is the impact of a 1.8 nm gate length (18A nanotechnology) for a source/drain channel in a FinFET device (size effect)? The diameter of a Si-atom is about 2 angstroms (~0.22 nm). This means that the channel of 1DEG between source and drain (*x*-axis, the orientation of current flow) is formed only by nine Si atoms with *x*-length and a certain width (*y*-axis, which is also confined by 3D oxide barriers). In this nanowire channel, the electrons of 1DEG will more likely be transported in quantum ballistic model instead of classical Drude model, and the classical 3D electron scatterings will be eliminated by quantum confinement in 1D ballistic transportation, resulting in a higher mobility of 1DEG for better on/off switch performance due to the greater quantum lifetime of the 1D ballistic channel. More, the spin-splitting energy of an electron can be clearly detected in the reduced dimensional electron systems (e.g., 2DEG, 1DEG, and 0DEG) of III-V compound semiconductors, as compared to Si-MOSFETs, due to the larger intrinsic spin–orbital interaction, which is a gate-controllable parameter causing a Rashba effect as opposed to a Dresselhaus effect [7,8,9]. In particular, if the spin-splitting energy is large enough to identify the quantum spin-entangled states, it can be prospectively applied to a spin-polarized semiconductor nano-device for a qubit of quantum computing [10]. To engineer the band profile of semiconductor nanomaterials for a particular function of a nano-device (e.g., nanoelectronic FETs, high-sensitive nano-detectors, high-performance micro-LEDs, or even highly efficient solar cells), one can select the host materials of IV-IV (e.g., graphene, SiC, SiGe), III-V (e.g., III-Arenites, III-Nitrides), or II-VI compounds (e.g., ZnO, ZnS, ZnSe, CdS, CdSe, CdTe, HgTe, etc.) by means of a top-down DUV lithography approach or by bottom-up self-assembling nanotechnology, as shown in this Special Issue.

The thermal energy of a free electron is another key factor to execute quantum devices, i.e., *E*th(T, *f*) = *f* × (½*k_B_T*) for *f*-degree of freedom (*f* = 3, 2, 1 for 3D, 2D, 1DEGs, respectively) at temperature T, and *k_B_* = 8.617 × 10^−5^ eV/K is Boltzmann constant (thermal effect). When the separation of quantized electron energy levels between first and second subbands (i.e., Δ*E*_12_ = *E*_2_ − *E*_1_) is comparable or smaller than thermal energy, it is appropriate to empirically evaluate the device characteristics of 2DEG using a classical Drude model at room temperature because the discrete quantized energy levels are thermally smeared to continuous states. However, the separation of discrete energy levels is inversely proportional to the size of the device channel and becomes very large in the reducing nanometer electron systems, and the separation of discrete energy levels (Δ*E*_12_) can be greater than the thermal energy of the electrons, particularly at low temperatures. As a result, thermal scattering between inter-subbands is forbidden and electron mobility is enhanced, demonstrating that quantum effects cannot be ignored. Therefore, we define a quantum performance indicator (QPI) as a ratio of the separation of quantized energy levels versus thermal energy, QPI = Δ*E*_12_/*E*th(T, *f*), to evaluate the quantum performance of a reduced dimensional nano-device at operation temperature T. For instance, the Δ*E*_12_ = 26.8 meV at GaAs/Al_0.3_Ga_0.7_As heterostructured QW [3] and *E*th(300 K, 2D) = 25.851 meV (working in water cooling), *E*th(77 K, 2D) = 6.635 meV (in liquid nitrogen cooling), and *E*th(4.2 K, 2D) = 0.362 meV (in liquid helium cooling) lead to QPI = 1.0, 4.0, and 74.0, respectively. The quantum efficiency of GaAs-QW can be amplified by 74 times simply by lowering the operation temperature down to 4.2 K. This is the reason why the quantum Hall effect or Shubnikov–de Haas effect can be easily observed in GaAs-QWs at low temperatures [3,4]. It is noted that the present commercial quantum computer with a superconducting qubit is operated at a temperature lower than 0.3 K (dilute He^3^–He^4^ refrigerator cooling). On the other hand, the wide-bandgap semiconductors (e.g., SiC, GaN, ZnO, …) provide very mature compounds for hosting the large separation of quantized electron energy levels (Δ*E*_12_), leading to the development of high-temperature, high-efficiency, and high-power optoelectronic devices. The self-assembled nanomaterials for GaN compounds have been developed using a bottom-up approach for nanometer quantum devices, including nano-rods [11], micro-pyramids, and micro-disks [12]; and their applications can cover not only high-power nano-electronics but also sustainable lighting sources for new-generation micro-LED displays [13,14,15,16,17]. Therefore, one can design a pioneer quantum device for a special function by engineering semiconductor nanomaterials with an optimized band profile of subband energy levels to evaluate QPI for its quantum performance prior to industrial manufacture.

## 2. Overview of Contributed Articles

In Contribution 1, the authors (Hua Li et al.) demonstrated the effective charge injection in MAPbI_3_ QD/TiO_2_ heterojunctions, and the size effect of the rate of charge transfer on the size of large (13.3 ± 1.5 nm), medium (11.3 ± 1.7 nm), and small (9.4 ± 1.3 nm) QDs to be 1.6 × 10^10^, 2.8 × 10^10^, and 4.3 × 10^10^ s^−1^, respectively. The result showed a potential application of nanomaterials, MAPbI_3_ QDs, in high-performance and high-efficiency photovoltaic devices.

In Contribution 2, the authors (Hao-Yu Hsieh et al.) studied the relaxation of an AlGaN strain on a GaN porous structure by comparing variations in the d-spacing of lattice constant among nine samples with overgrown AlGaN on GaN templates. They found the behavior of tensile strain reduction in AlGaN when a porous structure was created in the GaN layer, and the critical thickness of AlGaN was increased. They also proposed that AlGaN surface cracking on an epi-film can be eliminated if the GaN interlayer right above the porous structure is sufficiently thin.

In Contribution 3, the authors (Yu-Chung Lin et al.) fabricated ternary In_x_Ga_1−x_N multiple quantum wells (MQWs) on GaN/LAO microdisk substrates by PAMBE, and the PL spectrums of the MQWs showed that the major peaks of blue, green, and red lights for the growth temperature decreased from 720 °C to 670 °C. They established a thermodynamic mechanism to optimize the In_x_Ga_1−x_N multiple quantum wells for micro-LEDs. They also demonstrated that three different lighting micro-LEDs (red, green, and blue) can be made by one single material (In_x_Ga_1−x_N) on GaN-QW microdisks using self-assembling nanotechnology for optoelectronic applications, such as a full-color micro-LED display.

In Contribution 4, the author (Isaac Balberg) studied the dependencies of transient conductivity I(t) and the corresponding transient current characteristics on the size of nanocrystallites (NCs), such as CdSe and Si NCs. He showed that the glassy-like slow decay of I(t) is sensitive to the size, revealing the influence of quantum confinement (QC) and Coulomb blockade (CB) effects on the glassy features. He found that the main feature of glassy behavior is the nano-nature of NCs rather than defect-related recombination effects, as in bulk semiconductors. He suggested that in the glassy behavior of these nanosystems, the CB plays a similar role to the cage in a classical glass system with viscosity. From the I(t) analyses, he drew a conclusion that the observed I(t)s in electrical systems of glassy behavior should be interpreted with two different mechanisms: the minimization of energy that determines the glassy behavior and the minimization of energy dissipation that determines electrical transport. Based on the mechanisms, one can understand the observed I(t)s for nano-semiconductor and photoconductor systems, such as quantum dot and quantum well devices.

In Contribution 5, the authors (Jiaxing Li et al.) studied the whispering-gallery-mode (WGM) of a boron nitride (BN) microdisk cavity and showed that the BN microdisk cavity had a stable WGM in the ultraviolet band from 270 nm to 350 nm. They found that the size effect of individual nanoparticles in the microdisk, and the detection of a single nanoparticle with a size of 140 nm was achieved, offering a prospective application for a biomolecular sensor.

Contribution 6, the authors (Qinyue Sun et al.) proposed a micro-LED naked-eye 3D display system based on a double-layer metasurface thin film structure. They found a parallel light deflection metasurface with eight angles (in the range between approximately −45° and 45°), a high-efficiency directional emission light (greater than 80%) and low crosstalk. The pixel density of the monitor is as high as 605 PPI, and the peak values of the adjacent left-eye and right-eye views are 65 mm apart (about the distance between human eyes) to ensure the imaging requirement for a naked-eye 3D display, demonstrating the prospective potential of this application.

## 3. Summary

In this Special Issue, we offer a platform for researchers to present their findings on the engineering of semiconductor nanomaterials for optoelectronic applications, using a top-down DUV lithography approach or bottom-up self-assembling approach. We also define a quantum performance indicator (QPI) to evaluate in advance the quantum performance of reduced dimensional 2D/1D/0D nanomaterials for the device applications (e.g., nanometer FETs, high-sensitive nano-detectors, high-performance micro-LEDs, and high-efficient solar cells). Six articles were contributed to this Special Issue, as shown in the list, which cover “efficient charge transfer in MAPbI_3_ QDs/TiO_2_ heterojunctions for high-performance solar cells” (by Hua Li et al.), “AlGaN strain relaxation on GaN porous structure” (by Hao-Yu Hsieh et al.), “optimization of ternary In_x_Ga_1−x_N quantum wells on GaN microdisks” (by Yu-Chung Lin et al.), “glassy-like transients in semiconductor nanomaterials” (by Isaac Balberg), “nanoparticle detection in the ultraviolet region based on boron nitride microdisk” (by Jiaxing Li et al.), and “double-layer metasurface with micro-LED for 3D display” (by Qinyue Sun et al.). From the contributed articles, readers can learn about the latest achievements in the engineering of semiconductor nanomaterials (including: porous structures, quantum dots, microdisk-QWs, nanocrystallites, microdisk-cavity, and microdisk-displays, etc.), evaluate the perspective trends in the higher quantum-efficient QPI nano-devices for several applications, and estimate the difficulty of achieving the extreme size of semiconductor nanomaterials prior to industrial manufacture.
